# Enhanced Safety Surveillance of Influenza Vaccines in General Practice, Winter 2015-16: Feasibility Study

**DOI:** 10.2196/12016

**Published:** 2019-11-14

**Authors:** Simon de Lusignan, Ana Correa, Gaël Dos Santos, Nadia Meyer, François Haguinet, Rebecca Webb, Christopher McGee, Rachel Byford, Ivelina Yonova, Sameera Pathirannehelage, Filipa Matos Ferreira, Simon Jones

**Affiliations:** 1 University of Surrey Department of Clinical and Experimental Medicine Guildford United Kingdom; 2 GlaxoSmithKline Wavre Belgium; 3 Royal College of General Practitioners London United Kingdom; 4 NYU School of Medicine Department of Population Health New York, NY United States

**Keywords:** vaccines, safety management, medical records systems, computerized, drug-related side effects and adverse reactions, influenza, human, influenza vaccines, general practice, England

## Abstract

**Background:**

The European Medicines Agency (EMA) requires vaccine manufacturers to conduct enhanced real-time surveillance of seasonal influenza vaccination. The EMA has specified a list of adverse events of interest to be monitored. The EMA sets out 3 different ways to conduct such surveillance: (1) active surveillance, (2) enhanced passive surveillance, or (3) electronic health record data mining (EHR-DM). English general practice (GP) is a suitable setting to implement enhanced passive surveillance and EHR-DM.

**Objective:**

This study aimed to test the feasibility of conducting enhanced passive surveillance in GP using the yellow card scheme (adverse events of interest reporting cards) to determine if it has any advantages over EHR-DM alone.

**Methods:**

A total of 9 GPs in England participated, of which 3 tested the feasibility of enhanced passive surveillance and the other 6 EHR-DM alone. The 3 that tested EPS provided patients with yellow (adverse events) cards for patients to report any adverse events. Data were extracted from all 9 GPs’ EHRs between weeks 35 and 49 (08/24/2015 to 12/06/2015), the main period of influenza vaccination. We conducted weekly analysis and end-of-study analyses.

**Results:**

Our GPs were largely distributed across England with a registered population of 81,040. In the week 49 report, 15,863/81,040 people (19.57% of the registered practice population) were vaccinated. In the EPS practices, staff managed to hand out the cards to 61.25% (4150/6776) of the vaccinees, and of these cards, 1.98% (82/4150) were returned to the GP offices. Adverse events of interests were reported by 113 /7223 people (1.56%) in the enhanced passive surveillance practices, compared with 322/8640 people (3.73%) in the EHR-DM practices.

**Conclusions:**

Overall, we demonstrated that GPs EHR-DM was an appropriate method of enhanced surveillance. However, the use of yellow cards, in enhanced passive surveillance practices, did not enhance the collection of adverse events of interests as demonstrated in this study. Their return rate was poor, data entry from them was not straightforward, and there were issues with data reconciliation. We concluded that customized cards prespecifying the EMA’s adverse events of interests, combined with EHR-DM, were needed to maximize data collection.

**International Registered Report Identifier (IRRID):**

RR2-10.1136/bmjopen-2016-015469

## Introduction

### Background

The European Medicines Agency (EMA) released interim guidance on enhanced safety surveillance for seasonal influenza vaccines in August 2014 [1]. All Marketing Authorization Holders (MAHs) commercializing influenza vaccines in Europe must follow this guidance which sets out new standards for safety surveillance. Its goal is to rapidly detect, in near real-time early in the season, any significant increase in the frequency or severity of a defined list of adverse events of interest (AEIs). These AEIs can be local, systemic, or allergic reactions, indicating a potential or more serious risk.

For this request, the EMA defines 3 types of surveillance: (1) active surveillance, using existing methods of postauthorization surveillance; (2) enhanced passive surveillance (EPS), estimating vaccine usage rapidly and taking additional steps to facilitate passive adverse drug reactions (ADR) reporting of incidence of AEIs; and (3) electronic health record data mining (EHR-DM).

English general practice (GP) is a suitable setting to implement EPS and EHR-DM, as it has a registration-based list system with linked medical records, it has been highly computerized since 2004, and data extracted from these systems are widely used in research [2,3]. Furthermore, GPs are largely independent professional partnerships and make their own decision about which brand of influenza vaccine to purchase before the start of each influenza season [4]. Practices administer influenza vaccines to recommended groups, starting in September of each year [5].

### Objectives

In 2015-16, one of the major suppliers of influenza vaccines to UK health care was GSK (GlaxoSmithKline). Collaborating with the University of Surrey, GSK took the opportunity of the UK computerized infrastructure to implement EPS and EHR-DM with the aim to test the feasibility of using EPS and EHR-DM within GP to identify AEIs in subjects vaccinated with GSK’s and other influenza vaccine brands. An additional aim was to ascertain if using EPS in GP has any advantages over EHR-DM alone. The key components of this surveillance were as follows: (1) to provide a weekly estimation of vaccine coverage, by age, risk group, and vaccine brand; and (2) to estimate weekly AEI reporting rates among subjects vaccinated against seasonal influenza, by age, comorbidity, and brand from the GPs using the EHR-DM method, and those using the EPS system.

## Methods

### Setting

Following the request of EMA stating that MAHs must conduct enhanced seasonal influenza vaccines safety surveillance, GSK, together with the University of Surrey, launched a pilot safety surveillance study for GSK’s seasonal influenza vaccines. The University of Surrey has developed methods to extract timely surveillance data from GP. This system was developed to support the Royal College of General Practitioners (RCGP) Research and Surveillance Centre (RSC) weekly surveillance reports [6] and is capable of being adapted to support the EMA-specified surveillance (ClinicalTrials.gov number: NCT02567721).

We recruited 9 GPs spread across England, from varying types of locality (North and South, and urban and rural) and using different EHR systems (Egton Medical Information Systems) and The Phoenix Partnership). We compared the demographic characteristics—age, gender, ethnicity, and deprivation scores—using the Index of Multiple Deprivation, on the basis of each patient’s Lower Super Output Area (a small local geographical location [7,8]) of the registered population, with the national average data obtained from the Office for National Statistics [9].

### Design

This feasibility study period ran from International Organization for Standardization (ISO) week 35 to week 49 of 2015 (08/24/2015 to 12/06/2015).

As this was a feasibility study, there was no attempt to determine a priori the size of the study on the basis of power calculation. However, considering the average GP size of 7034 in England and Wales, it was expected that 9 GPs would provide a study population of 63,300. Ultimately, the population registered in the recruited practices was larger (N=81,040).

GPs were recruited in summer 2015; some months after they had selected their brand of vaccine for the coming influenza season. Out of the 9 practices recruited, 3 joined the EPS group and 6 joined the EHR-DM group. As this was a feasibility study, there was no attempt to randomize the practices into groups according to the type and brand of seasonal influenza vaccine used by the practices. Each group (EPS vs EHR-DM) was based on practices’ willingness to participate in the study.

The EPS group involved every patient who was vaccinated and received a *yellow card*; on the basis of the standard ADR cards used by the Medicines & Healthcare Products Regulatory Agency (MHRA) [10]. The patients were asked to complete a *yellow card* if they experienced an AEI in the 14-day postvaccination window; they were invited to return the card to their registered practice. The reported information was coded into the patient’s EHR by their GP and extracted weekly:

- EHR data mining: The EHR-DM group (n=6) had routine clinical data extracted from their EHR, pseudonymized and automatically sent to a secure sever on a weekly basis. In case of data extraction failure, a local data extraction was conducted using a Department of Health data extraction tool (MIQUEST—Morbidity Information Query and Export Syntax [11,12]). All practices in the EHR-DM group used a non-GSK influenza vaccine.
- Enhanced passive surveillance: The EPS group of practices (n=3) had their routine clinical data extracted from their EHR in the same way as the EHR-DM group. In addition, these practices also distributed a yellow card to patients receiving the seasonal influenza vaccine. Patients were asked to return the card to their own practice within 14 days. The information in the returned cards was then coded by practice staff into each patient’s EHR and then extracted in the same manner as the EHR-DM group. In this group, 2 practices used GSK influenza vaccine and 1 practice non-GSK vaccines.

All practices were given induction training and a preferred code list for the specific AEIs identified by EMA to facilitate standardized data coding. We created this list using an ontological approach [13,14]. We grouped these conditions into body system categories: respiratory, gastrointestinal, fever, sensitivity and anaphylaxis, rash, general symptoms, neurological, musculoskeletal, and local reactions (Multimedia Appendix 1). We provided GPs a full code list and a screen-side prompt list.

We requested that, when a patient presented with one of these conditions, they should use the recommended code (as per the instruction sheet). We advised GPs that GPs should code the conditions they felt a patient was reporting accurately. We stressed that the overarching purpose was not to purposively make any causal link between vaccination and a given condition, particularly for common conditions, such as cold and headache. However, they were reminded that they should report, in parallel to this study, any serious or important events on the basis of their professional judgment through the standard MHRA reporting system as per local regulation.

### Study Measures

The primary outcome measure was to report AEI frequencies among influenza-vaccinated subjects and observe any discrepancies in these frequencies in the EPS versus EHR-DM group and GSK versus non-GSK vaccines. To identify event date, we used the event date for the actual episode, not the recorded date.

- Estimation of vaccine uptake rates by age-band, risk group, and vaccine brand: We classified patients as vaccinated when a prescription or administration code for an influenza vaccine was recorded in the patient’s EHR. Whichever had the earliest date was taken as the vaccine administration date; prescription issue dates often lag behind the administration dates.
- Estimation of AEI rates by age, risk group, vaccination status, and vaccine brand: We included presentations up to 14 days, notwithstanding the EMA recommendations being for 7 days, to allow for any lag between experiencing an AEI and obtaining an appointment to see a GP or to return the yellow card. Both EHR and yellow card data were used to estimate AEIs in the EPS practices. We predicted that the yellow cards would increase AEI reporting; therefore, EPS practices should have a larger percentage of AEIs recorded.
- To observe the reporting trend from a broader pool of GPs, we also extracted the AEIs from the RCGP RSC Network: We focused on records of vaccinated subjects only, using data for the same weeks (35-49) in 2015. We did not include any events before vaccination for the 9 study practices or the RCGP RSC network. Analysis was concentrated on AEIs at any time after vaccination (not just in the 14-day window after vaccination).

Throughout the study, we produced weekly reports of AEIs. At the end of the study, we produced an end-of-season report. The end-of-season report enabled us to identify whether the approach was appropriate to adequately capture the vaccination uptake, to enhance the collection of AEs, easily transcribe the events reported from the yellow cards to the electronic system, more comprehensively capture the AEIs experienced, and extract them on an ongoing basis in a near real-time manner.

- Weekly reports: We produced weekly reports of the incidence of AEIs in influenza-vaccinated patients. We also reported cumulative vaccination rates. These weekly reports were intended to be produced the week following data recording. These reports provided practices feedback about the rate of AEIs recorded in their practice to encourage data recording. We reported the cumulative data adding the information as the data collection was progressing with patients registered until week 49 with the objective to assess the data in a near real-time manner. We included all patients with a valid registration (defined as fully registered with a valid registration) on the Friday of the week, before data extraction took place.
- End of study period analysis: We conducted an analysis at the end of the study period between weeks 35 and 49. We included patients registered with the pilot practices throughout the whole observation period with medical records valid 12 months before the start of the study; this objective being to ensure further that we had sufficient medical history about any long-term condition that might affect their priority for influenza vaccination.

Our established data extraction method means that only coded data (Read code version 2 and CTV3) [15] were used to collect the relevant information. Free text was not extracted as it might include patient identifiable information. We excluded people with a code that indicated that they opted out of sharing data; estimated at around 1.25% of the registered population [16]. Data were pseudonymized as close to source as possible and encrypted.

### Analyses

For all AEIs, we report the rate and the 95% CI, using the critical binomial function in Microsoft Excel [17].

## Results

### Setting

Our practices were largely distributed across England, with most in the Midlands and East National Health Service (NHS) Region (NHS Regions: North, n=3; Midlands and East, n=5; and South, n=1). Practices were mainly from urban areas (rural, n=3; and urban, n=6). In the week 49 weekly report (data extracted in a near real-time manner), the overall population registered in the 9 GP practices was 81,040. The end-of-study cohort population was 71,407 (owing to restriction of longer registration as described earlier).

**Figure 1:**
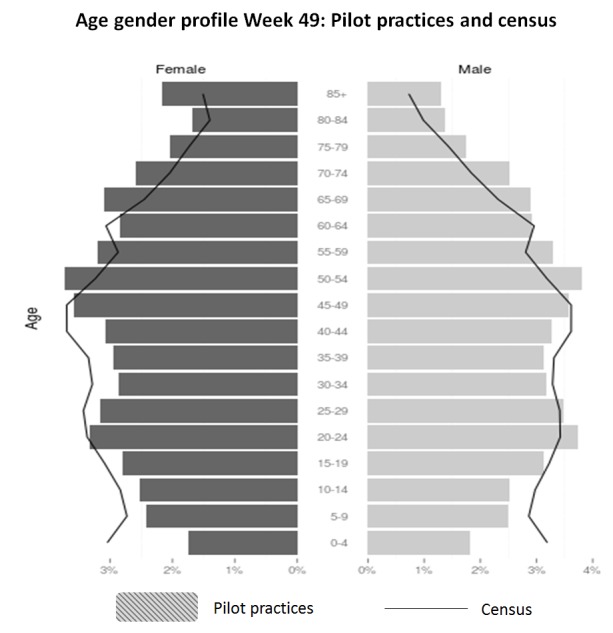
Age and sex distribution of pilot practices compared against the census.

In the week 49 data extraction, the practice populations had a female to male ratio of 49.76% (40, 323/81,040) to 50.24% (40,717/81,040. The proportion of people aged 20 to 24 years and over 50 years was above the national average (Figure 1). The study population had a higher proportion of people of white ethnicity recorded 92.57% (38,154/41,218 compared with 85.42% nationally; (45,281,142/53,012,456). The study population was less deprived than the national average, with almost three-fourths 72.20% (56,545/78,322) of the population in the least deprived half (Multimedia Appendix 2).

When comparing the EHR-DM practices and the EPS practices using the end-of-season dataset, the EPS practices were slightly older, the proportion of 65 years and over was 21.64% (n=9842/45,519) in EHR-DM compared with 25.44% (6586/25,888) in EPS (Table 1). The EHR-DM practices also had a smaller proportion of at-risk patients at 40.43% (18,403/45,519) compared with 45.21% (11,703/25,888) in the EPS practices.

**Table 1:** Summary of practice population from end of season cohort.

Variables			Vaccination status (cohort)	
			EHR-DM^a^	EPS^b^
Number of practices			6	3
**Age group (years)**				
	**<5**			
		n (%)	1097 (2.41)	535 (2.07)
		LCI^c^, %	2.27	1.90
		UCI^d^, %	2.55	2.24
	**5-14**			
		n (%)	4725 (10.38)	2638 (10.19)
		LCI, %	10.10	9.82
		UCI, %	10.66	10.56
	**15-64**			
		n (%)	29,855 (65.59)	16,129 (62.30)
		LCI, %	65.15	61.71
		UCI, %	66.03	62.89
	**65+**			
		n (%)	9842 (21.62)	6586 (25.44)
		LCI, %	21.64	24.91
		UCI, %	22.00	25.97
**Gender**				
	**Male**			
		n (%)	23,035 (50.64)	12,853 (49.65)
		LCI, %	50.19	49.04
		UCI, %	51.10	50.26
	**Female**			
		n (%)	22,466 (49.36)	13,035 (50.35)
		LCI, %	48.90	49.74
		UCI, %	49.81	50.96
**Risk group**				
	**Specific risk group**			
		n (%)	18,403 (40.43)	11,703 (45.21)
		LCI, %	39.98	44.60
		UCI, %	40.88	45.81

^a^EHR-DM: electronic health record data mining.

^b^EPS: enhanced passive surveillance.

^c^LCI: lower confidence interval.

^d^UCI: upper confidence interval.

### Measures

**Figure 2:**
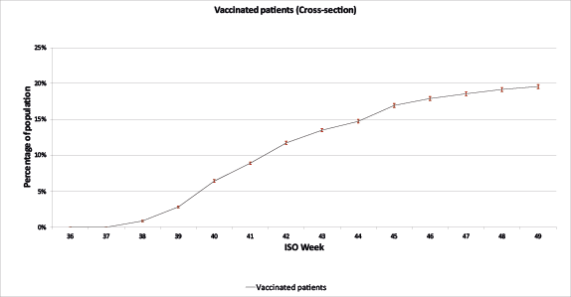
Cumulative vaccines.

Most vaccinations took place between weeks 39 and 45 (Figure 2). From the weekly cumulative data extractions (weeks 35-49), 19.57% (15,863/81,040) of the registered practice population overall and 57.48% (9969/17,344) of people aged 65 years or older were vaccinated (Table 2).

**Table 2:** Summary of vaccinations from weekly data extractions by risk group.

Variables			Vaccination status (weekly cumulated data)			
			EHR-DM^a^ vaccinated (Non-GSK)	EPS^b^		
				Vaccinated (Non-GSK)	Vaccinated (GSK)	Vaccinated (All)
**Number of practices**			**6**	**1**	**2**	**3**
	n (%)		8640 (16.62)	3607 (12.41)	3616 (12.44)	7223 (24.85)
	LCI^c^, %		16.30	12.03	12.06	24.35
	UCI^d^, %		16.95	12.79	12.82	25.35
**Risk group**						
	**Specific risk group**					
		n (%)	7422 (36.26)	2967 (22.66)	3301 (25.21)	6268 (47.88)
		LCI, %	35.60	21.94	24.47	47.02
		UCI, %	36.92	23.38	25.96	48.73
	**Under 4 years old**					
		n (%)	277 (14.70)	113 (10.89)	53 (5.11)	166 (15.99)
		LCI, %	13.11	9.06	3.85	13.78
		UCI, %	16.30	12.81	6.45	18.21
	**65 years or older**					
		n (%)	5198 (49.98)	2130 (30.67)	2641 (38.03)	4771 (68.71)
		LCI, %	49.02	29.59	36.90	67.61
		UCI, %	50.94	31.75	39.17	69.80

^a^EHR-DM: electronic health record data mining.

^b^EPS: enhanced passive surveillance.

^c^LCI: lower confidence interval.

^d^UCI: upper confidence interval.

#### Enhanced Passive Surveillance Practices

In the 3 practices that conducted *yellow card*–based surveillance, the staff managed to hand out the cards to 61. 24% (4150/6776) of the vaccinees, and of these cards, 1.97% (82/4150) were returned to the GP offices, which represented 1.21% (82/6776) of the vaccinated population covered by this surveillance system.

Some practices found it challenging to interpret and link the free-text comments on the cards to the specified AEI codes. With this respect, 1 practice faced some challenges to record all the information on the returned cards into the practice EHR system, the main reason being the difficulty to transcribe the events reported in the yellow cards to the electronic system.

Of those vaccinated (19.57% (15,863/81,040) of the population); 77.20% (12,247/15,863) were vaccinated using a non-GSK vaccine. The non-GSK vaccines that patients were administered were manufactured by Astra Zeneca, Sanofi Pasteur Europe, Seqirus Vaccines Limited, and Abbot Biologicals.

#### Adverse Events of Interest

From the weekly cumulative data extractions (weeks 35-49), the rates of AEIs in the 14-days postvaccination between GSK and non-GSK vaccines were similar: non-GSK=3.02% (370/12,247; 95% CI 2.72-3.33) and GSK=2.63% (95/3616; 95% CI 2.13-3.15) (Table 3). The most common AEIs presenting were respiratory, fever, and musculoskeletal symptoms (Multimedia Appendix 3).

The GSK vaccine had fewer AEI presentations of respiratory symptoms and fever but more musculoskeletal symptoms. The rates of these AEIs were as follows:

- Respiratory—0.96% (95% CI 0.79%-1.14%, 118/12,247) for non-GSK and 0.88% (95% CI 0.58%-1.19% 32/3616) for GSK vaccine.
- Fever—0.79% (95% CI 0.64%-0.96%, 97/12,247) for non-GSK and 0.66% (95% CI 0.41%-0.94%, 24/3616) for GSK vaccine.
- Musculoskeletal conditions—0.51% (95% CI 0.39%-0.65%, 63/12,247) for non-GSK and 0.91% (95% CI 0.61%-1.24%,33/3616) for GSK vaccine (Multimedia Appendix 3).

The highest rate of AEIs was identified in adults aged 15 to 64 years for GSK vaccines (3.2%; 25/781) (Table 3).

**Table 3:** Summary of adverse events of interest from weekly data extractions.

Any AEI^a^			Vaccination status (weekly cumulated data)			
			EHR-DM^b^ vaccinated (Non-GSK)	EPS^c^		
				Vaccinated (Non-GSK)	Vaccinated (GSK)	Vaccinated (All)
**Number of practices**			**6**	**1**	**2**	**3**
**Total**						
	n (%)		339 (3.92)	31 (0.43)	95 (1.32)	126 (1.74)
	LCI^d^, %		3.52	0.29	1.07	1.45
	UCI^e^, %		4.34	0.58	1.58	2.05
**Age groups (years)**						
	**<5**					
		n (%)	15 (5.42)	1 (0.60)	1 (0.60)	2 (1.20)
		LCI, %	2.12	0.00	2.31	0.00
		UCI, %	6.06	7.50	6.15	5.66
	**5-14**					
		n (%)	24 (3.37)	1 (0.18)	1 (0.18)	2 (0.36)
		LCI, %	0.98	0.00	1.32	0.00
		UCI, %	2.50	5.88	2.89	5.26
	**15-64**					
		n (%)	111 (4.53)	12 (0.69)	25 (3.20)	38 (2.20)
		LCI, %	3.11	2.05	2.95	1.73
		UCI, %	4.46	4.48	4.19	3.93
	**65+**					
		n (%)	189 (3.64)	17 (0.36)	67 (1.40)	84 (1.76)
		LCI, %	2.34%	1.95%	2.27%	1.67%
		UCI, %	3.11%	3.16%	2.99%	2.76%
**Risk group**						
	**Any risk group**					
		n (%)	293 (3.95)	29 (0.46)	87 (1.39)	116 (1.85)
		LCI, %	3.52	0.30	1.10	1.53
		UCI, %	4.39	0.64	1.69	2.19

^a^AEI: adverse events of interest.

^b^EHR-DM: electronic health record data mining.

^c^EPS: enhanced passive surveillance.

^d^LCI: lower confidence interval.

^e^UCI: upper confidence interval.

#### Hospital Admissions

We explored hospital admission data recorded in the practice EHR. The rate of admissions of vaccinees with an AEI was 2.50% (44/1,761; 95% CI 1.8-3.2).

Those receiving GSK vaccine had a numerically lower rate of hospital admission, although small numbers were reported. For example, 2% (2/92; 95% CI 2.2%-3.3%) of people vaccinated with GSK influenza vaccine were admitted to hospital with an AEI in the 14 days following vaccination, while in the non-GSK vaccines group, the rate was 3.9% (13/333; 95% CI 1.8%-2.1%).

There were timeliness, completeness, and accuracy issues with the retrieved hospital data. The hospital admission data and diagnoses took up to 10 to 20 days to be recorded in the practices’ EHR and, in some cases, up to 42 days.

### Analyses

#### End-of-Season and Weekly Comparison

The AEIs reported in the end of season and weekly extractions were comparable. For example, focusing on GSK vaccines, for any adverse event, the end-of-study cohort had an AEI recording of 92/3434 (2.68%) and weekly report of 95/3616 (2.63%). For GSK vaccines, the largest difference in AEIs was for any sensitivity or anaphylaxis (end of season: n=0, 0%; cohort: n=1, 0.03%).

#### Data Extraction From the Royal College of General Practitioners’ Research and Surveillance Centre

Overall, the study data were comparable with that of the rest of the RCGP RSC (see Multimedia Appendix 3). This suggests that the AEIs reporting patterns from the 9 GPs recruited and the RCGP RSC were similar and that the data extraction approach was valid.

Timeliness was comparable with RCGP RSC. Data were extracted each week using the same automated system. However, 2 practices required local extracts (carried out where the remote systems installation failed); these 2 practices had delays of up to 2 weeks, particularly early in the observation period.

The RCGP RSC rate of overall influenza vaccine uptake (excluding the study practices) is 21.80% (238,519/1,094,352); the overall rate of these 9 practices, using the end-of-study cohort data, was 20.73% (14,801/71,407) suggesting a good completeness of the study data for vaccine uptake.

## Discussion

### Principal Findings

This study showed that using GP data for EHR-DM was a feasible method of near real-time surveillance. This was demonstrated by the timeliness of data extraction and the validity of the data.

The use of *yellow cards* (EPS) to enhance surveillance was not successful as there was only a 1.97% (82/4150) return rate; and this small additional return rate did not yield useful additional information. However, there were only 3 practices in this group.

Rates of AEI (within 14 days postvaccination) in the vaccinated group were around 3% (3.02% 333/11,367 for non-GSK and 2.63% 92/3434 for GSK) with around 1% (150/15,863) of AEIs were respiratory conditions. The differences in rates were not statistically significant, and no safety concerns were raised during the weekly assessment or at the end of the study period.

### Implications of Findings

The study demonstrated the feasibility of setting up a network that could, with further refinement, rapidly detect potential safety concerns, allowing prompt investigation if deemed appropriate [18], originating from a significant change in AEIs associated with influenza vaccination using EHR-DM observing, for example, trends from week to week.

This network still needs additional adaptations to be worked out in the next phase of its implementation. For example, further thought needs to be given regarding improving the return rate of *yellow cards*. To improve the return rate, any future ADR card should ideally have predefined categories that patients can tick if present (or record if no AEI is experienced following vaccination), to standardize coding into the EHR.

To harmonize and standardize the Enhanced Safety Surveillance in Europe, one recommendation could be to establish a network of organizations involved in EHR data mining in real time, supplemented with vaccination-customized ADR cards (ie, *yellow card*s in this study) reporting scheme, with criteria defining the prerequisites of data quality. However, a *yellow card* is not a good long-term choice of color or label. In the United Kingdom, *yellow card*s are developed by the MHRA and are generally completed when a GP suspects an ADR related to the vaccination. In this study, the approach was to collect as comprehensively as possible AEIs, prespecified by the EMA, regardless of the possible causal association with immunization.

Another potential way to collect AEI following vaccination could be to develop a mobile phone application to collect events in a more comprehensive manner. This approach could be an interesting approach to overcome the suboptimal return. Future research could investigate the feasibility of designing such an app to collect this information in a more reliable manner.

### Generalizability

A near real-time enhanced brand-specific surveillance network could produce weekly reports of AEIs to meet the EMA requirements. The data extraction element of the project could readily be extended; the *yellow card* scheme could be refined and expanded. Introducing an ADR card as part of the process may be an optimal and sustainable way to stimulate reporting of AEIs from those with a lower propensity to consult (ie, for mild events).

Data collection over several seasons may be required to achieve a better understanding of background rates of AEIs. A particular challenge is that people immunized in the first weeks of immunization may differ from those immunized in subsequent weeks due to annual recommendations to promptly vaccinate the more vulnerable patients early in the season.

It is feasible to set up a weekly reporting, using an enhanced passive and EHR-DM surveillance system, to detect EMA-specified AEIs across specific brands; though further refinements are needed before such a system can be fully operationalized.

### Limitations

Data were inevitably passive surveillance data, largely derived from medically attended events for those who consult. Considering that some groups have a lower propensity to consult (eg, men [19]), this is a likely confounding factor. Future EPS may need to be designed to be more inclusive of less represented groups; for instance, using customized ADR cards to continuously enhance the reporting.

A further limitation of our design was the potential bias in the practice selection for EPS, which was conditional on the willingness of practice to participate in the study and hand out, collect, and code any data on the *yellow cards*. We drew conclusions about the unsuitability of the yellow card on basis of data from 3 practices, not selected at random. Return rates were low and what was written on the cards was challenging to transcribe and thus to code consistently.

In addition, in the United Kingdom, vaccines are preferentially recommended for different age and risk groups which preclude any possibility to systematically compare the findings from different vaccine brands. In addition, the sample for future studies could have improved representativeness by recruiting more inner-city practices. Our study practice populations were less ethnically mixed and less deprived than the English national average, likely due to a lack of inner-city practices.

Our remote data extraction system was reasonably reliable, although it had gaps. It is challenging to use a local extract system in a timely way, although it did fill gaps in our data. One other challenge was to properly analyze all the collected data to allow near real-time assessment. In the longer term, comparing rates with historic data may be an additional option to have more insights on baseline rates as a basis for comparison with findings in the current season. For this study, we had a lack of historical background rates of AEI in the same population or availability of an appropriate comparison group. In future studies, we should either compare rates with AEIs derived from the RCGP or from previous years of these studies, assuming that the study design and the approach remain overall unchanged.

A limitation of our design was training GPs in coding. While this should help with data within the season, it has the potential to produce biased results when comparing with historical data when no training was given. Furthermore, the preferred code list given to practices (Multimedia Appendix 1) notes that these codes should be used for adverse events postvaccination. In this respect, it does not appear to use as baseline for comparison historical data where no specific training was provided.

### Conclusions

Overall, this study showed that using GP data was a feasible method for enhanced near-real time surveillance in terms of EHR-DM. However, the use of *yellow cards* (EPS) in GP did not capture a significant amount of additional data. There are many lessons learnt from this initial study, and these are reflected in the limitations of the design and study approach. Future enhanced surveillance should focus on ways to improve and standardize AEIs reporting, data collection, and extraction.

## Appendix

Multimedia Appendix 1 Age and sex distribution of pilot practices compared against the census.

Multimedia Appendix 2 Distribution of deprivation within pilot practices in comparison to the census.

Multimedia Appendix 3 Tabular summary of AEIs.

## Abbreviations

ADR: adverse drug reactions
AEIs: adverse events of interest
EHR-DM: electronic health record data mining
EMA: European Medicines Agency
EPS: enhanced passive surveillance
GP: general practice
MAHs: Marketing Authorization Holders
MHRA: Medicines & Healthcare Products Regulatory Agency
NHS: National Health Service
RCGP: Royal College of General Practitioners
RSC: Research and Surveillance Centre
