# Supplementary material for: Enhanced Safety Surveillance of Influenza Vaccines in General Practice, Winter 2015-16: Feasibility Study
Source: JMIR Public Health Surveill. 2019 Nov 14;5(4):e12016. doi: 10.2196/12016 (PMC6913774; doi:10.2196/12016)
Supplement: Multimedia Appendix 1 [file publichealth_v5i4e12016_app1.pdf]

European Medicines Agency (EMA) post-authorisation safety study of influenza vaccine – Preferred code list

If a patient presents with adverse events post-vaccination (up to 14 days after), please code (ideally as a problem) as any of the following:

EMA surveillance condition	Read Code (5 Byte)	Read Code (CTV3)	Description	Notes
**Respiratory/Miscellaneous**				
Conjunctivitis	F4C0.	XE16X	Acute conjunctivitis	
Rhinorrhoea	1C83.	XM00h	Rhinorrhoea	
Nasal congestion	H1y1z	X77Gp	Nasal airway obstruction	
Epistaxis	R047.	Xa96W	Epistaxis	
Coryza	H00..	XE0Xl	Acute coryza	
Cough	171..	XM0Ch	Cough	
Oropharyngeal pain	1922.<br>1CB3.	1922.<br>1CB3.	Sore mouth/Throat pain	
Hoarseness	1CA2.	1CA2.	Hoarse	
Wheezing	1737.	XE0qs	Wheezing	
**Gastrointestinal**				
Decreased appetite	R0300	XM07Y	Loss of appetite	
Nausea	198..	X75qw	Nausea	
Vomiting	199..	XE0rA	Vomiting	
Diarrhoea	19F..	19F2.	Diarrhoea	
**Fever/pyrexia**				
Fever	165..	X76Dl	Fever symptoms	
Mild fever (<38.5° C rectal)<br>Moderate fever (38.6-39.5°C)<br>High fever (>39.5°C)	2E3..	2E3..	O/E – Temperature level	Please include level of temperature, to help us classify the fever
**Sensitivity/anaphylaxis**				
Hypersensitivity reactions	SN52.	Xa5uf	Adverse drug reaction/Vaccine allergy	
Anaphylactic reactions	SN501	X70vr	Drug-induced anaphylaxis	
Facial oedema	16J5.	Xa0ls	Facial swelling	
Local erythema	SP3y5	X75ty	Erythema at injection site	
**Rash**				
Rash	M130.	X50Ge	Drug-induced rash	
Generalised rash	2I14.	XM07J	Rash	
Local erythema	SP3y5	X75ty	Erythema at injection site	
**General non-specific symptoms**				
Irritability	225A.	225A.	O/E - Irritable	
Drowsiness	1B67.	XM06R	Drowsiness	
Fatigue	168..	1682.	Fatigue	
**Neurological**				
Peripheral tremor	1B22.	XE0rn	Tremor	
Guillain-Barre Syndrome (GBS)	F3700	F3700	Guillain-Barre Syndrome	
Seizure/ Febrile convulsions	1B64.<br>1B6B.	XaDbE<br>XM03l	Convulsion/Febrile convulsion	
Headache	1B1G.	XM0CV	Headache	
**Musculoskeletal**				
Muscle aches/ myalgia	N2410	X75rs	Myalgia	
Arthropathy	N037.	X701f	Post-immunisation arthropathy	

N.B.: In coding these conditions there is **no assumption about causation**; this can only come from advanced analytics.

UNIVERSITY OF SURREY

Principal Investigator Professor Simon de Lusignan

Practice Liaison Officer Ivelina Yonova (i.yonova@surrey.ac.uk)
