# Supplementary figures and images for: Enhanced Safety Surveillance of Influenza Vaccines in General Practice, Winter 2015-16: Feasibility Study
Source: JMIR Public Health Surveill. 2019 Nov 14;5(4):e12016. doi: 10.2196/12016 (PMC6913774; doi:10.2196/12016)

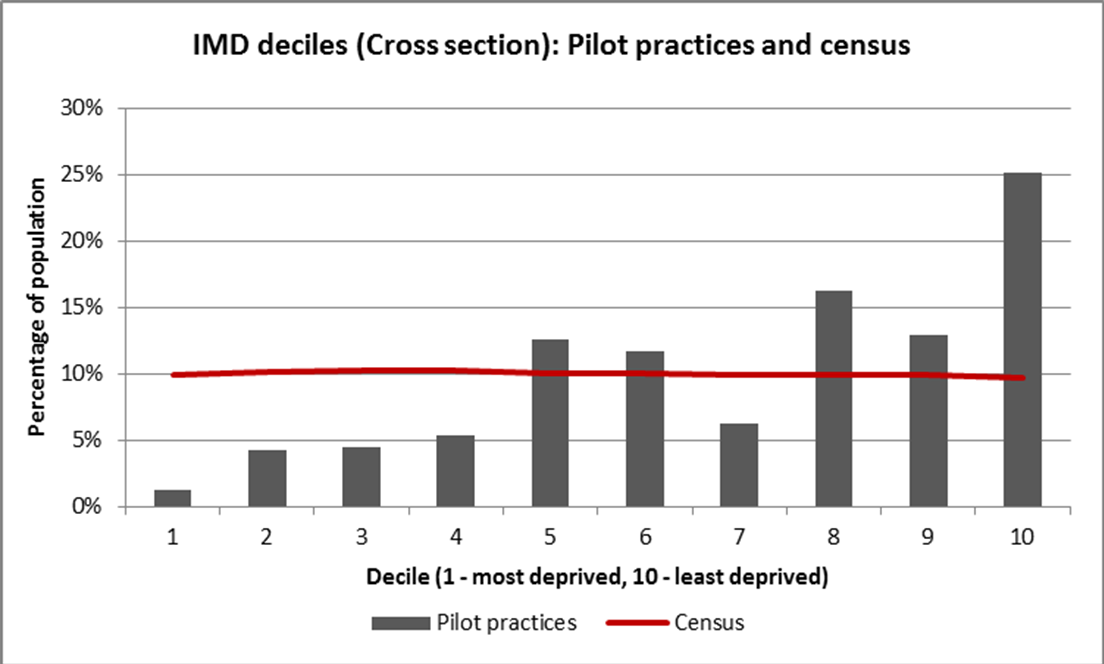

Supplement: Multimedia Appendix 2 [file publichealth_v5i4e12016_app2.png]
