# Supplementary material for: Enhanced Safety Surveillance of Influenza Vaccines in General Practice, Winter 2015-16: Feasibility Study
Source: JMIR Public Health Surveill. 2019 Nov 14;5(4):e12016. doi: 10.2196/12016 (PMC6913774; doi:10.2196/12016)
Supplement: Multimedia Appendix 3 [file publichealth_v5i4e12016_app3.pdf]

Supplementary Table 1 – Tabular summary of AEIs

		AEI (End of study period cohort) AEI within 14-day post-vaccination		AEI (Weekly report data) AEI within 14-day post-vaccination	
**System**		*Vaccinated (Non-GSK)*	*Vaccinated (GSK)*	*Vaccinated (Non-GSK)*	*Vaccinated (GSK)*
**No. of practices**	**n**	*7*	*2*	*7*	*2*
Any Adverse Event	**n**	333	92	370	95
	**%**	2.93%	2.68%	3.02%	2.63%
	**LCI**	2.62%	2.15%	2.72%	2.13%
	**UCI**	3.25%	3.23%	3.33%	3.15%
Any respiratory / miscellaneous	**n**	108	31	118	32
	**%**	0.95%	0.90%	0.96%	0.88%
	**LCI**	0.77%	0.61%	0.79%	0.58%
	**UCI**	1.13%	1.22%	1.14%	1.19%
Any gastrointestinal	**n**	24	2	31	2
	**%**	0.21%	0.06%	0.25%	0.06%
	**LCI**	0.13%	0.00%	0.17%	0.00%
	**UCI**	0.30%	0.15%	0.34%	0.14%
Any fever / pyrexia	**n**	87	23	97	24
	**%**	0.77%	0.67%	0.79%	0.66%
	**LCI**	0.61%	0.41%	0.64%	0.41%
	**UCI**	0.93%	0.96%	0.96%	0.94%
Any sensitivity / anaphylaxis	**n**	5	0	6	1
	**%**	0.04%	0.00%	0.05%	0.03%
	**LCI**	0.01%	N/A	0.02%	0.00%
	**UCI**	0.09%	N/A	0.09%	0.08%
Any rash	**n**	24	7	24	7
	**%**	0.21%	0.20%	0.20%	0.19%
	**LCI**	0.13%	0.06%	0.12%	0.06%
	**UCI**	0.30%	0.38%	0.28%	0.36%
Any general symptom	**n**	23	8	23	8
	**%**	0.20%	0.23%	0.19%	0.22%
	**LCI**	0.12%	0.09%	0.11%	0.08%
	**UCI**	0.29%	0.41%	0.27%	0.39%

Any neurological	n	31	6	34	6
	%	0.27%	0.17%	0.28%	0.17%
	LCI	0.18%	0.06%	0.19%	0.06%
	UCI	0.37%	0.32%	0.38%	0.30%
Any musculoskeletal	n	54	32	63	33
	%	0.48%	0.93%	0.51%	0.91%
	LCI	0.35%	0.61%	0.39%	0.61%
	UCI	0.61%	1.25%	0.65%	1.24%
Any local symptom	n	3	6	3	6
	%	0.03%	0.17%	0.02%	0.17%
	LCI	0.00%	0.06%	0.00%	0.06%
	UCI	0.06%	0.32%	0.06%	0.30%

Table 2 – Comparison of end of vaccination season rates with RCGP RSC network

		Vaccination status			
		End of study period cohort		RCGP RSC network 2014	
**No. of practices**		*9*		*124*	
		Vaccinated	*Non vaccinated*	*Vaccinated*	*Non vaccinated*
Total	n	14801	56606	227496	817982
	%	20.73%	79.27%	21.76%	78.25%
	LCI	20.43%	78.97%	21.68%	78.17%
	UCI	21.03%	79.57%	21.84%	78.33%

		AEI at any time	
		End of study period cohort	RCGP RSC network 2014
**AEI category**		*Vaccinated*	*Vaccinated*
**Any AEI**	**n**	1761	32062
	**%**	11.90%	14.09%
	**LCI**	11.38%	13.95%
	**UCI**	12.42%	14.24%
**Any Respiratory**	**n**	652	11729
	**%**	4.41%	5.16%
	**LCI**	4.08%	5.07%
	**UCI**	4.74%	5.25%
**Any Gastrointestinal**	**n**	201	3011
	**%**	1.36%	1.32%
	**LCI**	1.18%	1.28%
	**UCI**	1.55%	1.37%

**Any Fever / Pyrexia**	**n**	438	8259
	**%**	2.96%	3.63%
	**LCI**	2.69%	3.55%
	**UCI**	3.24%	3.71%
**Any Sensitivity / Anaphylaxis**	**n**	16	362
	**%**	0.11%	0.16%
	**LCI**	0.06%	0.14%
	**UCI**	0.16%	0.18%
**Any Rash**	**n**	150	2845
	**%**	1.01%	1.25%
	**LCI**	0.86%	1.21%
	**UCI**	1.18%	1.30%
**Any General**	**n**	126	2051
	**%**	0.85%	0.90%
	**LCI**	0.71%	0.86%
	**UCI**	1.00%	0.94%
**Any Neurological**	**n**	133	2300
	**%**	0.90%	1.01%
	**LCI**	0.75%	0.97%
	**UCI**	1.05%	1.05%
**Any Musculoskeletal**	**n**	357	7265
	**%**	2.41%	3.19%
	**LCI**	2.17%	3.12%
	**UCI**	2.66%	3.27%
**Any Local**	**n**	12	3
	**%**	0.08%	0.00%
	**LCI**	0.04%	0.00%
	**UCI**	0.13%	0.00%

Table 3 – Summary of vaccinations from end of study cohort by risk group.

	Vaccination status (Cohort)							
	EHR-DM		EPS		EPS		EPS	
	Vaccinated (Non-GSK)		*Vaccinated (Non-GSK)*		*Vaccinated (GSK)*		*Vaccinated (All)*	
**No. of practices**	*6*		*1*		*2*		*3*	
**Total**	8025		3342		3434		6776	
	17.63%		12.91%		13.26%		26.17%	
	17.28%	17.98%	12.50%	13.32%	12.85%	13.68%	25.64%	26.71%
	**Vaccination status (Cohort)**							
**Risk Group**	*Vaccinated (Non-GSK)*		*Vaccinated (Non-GSK)*		*Vaccinated (GSK)*		*Vaccinated (All)*	
Any risk group	6945		2740		3154		5894	
	37.74%		23.41%		26.95%		50.36%	

	37.04%	38.44%	22.64%	24.18%	26.15%	27.75%	49.46%	51.27%
Under 4 years old	236		94		40		134	
	21.51%		17.57%		7.48%		25.05%	
	19.14%	23.97%	14.39%	20.93%	5.42%	9.72%	21.50%	28.79%
Over 65 years old	4905		1985		2562		4547	
	49.84%		30.14%		38.90%		69.04%	
	48.85%	50.82%	29.03%	31.25%	37.73%	40.09%	67.92%	70.15%

Table 4 – Summary of AEIs from end of study cohort.

	Vaccination status (Cohort)							
**Any AEI**	**EHR-DM**		**EPS**		**EPS**		**EPS**	
	Vaccinated (Non-GSK)		***Vaccinated (Non-GSK)***		***Vaccinated (GSK)***		***Vaccinated (All)***	
**No. of practices**	***6***		***1***		***2***		***3***	
**Total**	302		31		92		123	
	3.50%		0.46%		1.36%		1.82%	
	3.10%	3.90%	0.31%	0.62%	1.09%	1.64%	1.51%	2.14%
	**Vaccination status (Cohort)**							
**Age Group**	***Vaccinated (Non-GSK)***		***Vaccinated (Non-GSK)***		***Vaccinated (GSK)***		***Vaccinated (All)***	
<5yr	12		1		1		2	
	5.08%		0.75%		0.75%		1.49%	
	2.54%	8.05%	0.00%	2.24%	0.00%	2.24%	0.00%	3.73%
5-14yrs	18		1		1		2	
	2.78%		0.19%		0.19%		0.38%	
	1.54%	4.17%	0.00%	0.57%	0.00%	0.57%	0.00%	0.95%
15-64yrs	102		12		25		37	
	4.56%		0.76%		1.59%		2.36%	
	3.71%	5.46%	0.38%	1.21%	1.02%	2.23%	1.66%	3.12%
65+ yrs	170		17		65		82	
	3.47%		0.37%		1.43%		1.80%	
	2.96%	4.00%	0.20%	0.57%	1.10%	1.78%	1.43%	2.20%
	**Vaccination status (Cohort)**							
**Risk Group**	***Vaccinated (Non-GSK)***		***Vaccinated (Non-GSK)***		***Vaccinated (GSK)***		***Vaccinated (All)***	
Any risk group	266		29		84		113	
	3.83%		0.49%		1.43%		1.92%	
	3.38%	4.29%	0.32%	0.68%	1.14%	1.73%	1.58%	2.27%
